# Investigations on the Influence of Floor Design on Dirtiness and Foot Pad Lesions in Growing Rabbits

**DOI:** 10.3390/ani9060354

**Published:** 2019-06-14

**Authors:** Till Masthoff, Steffen Hoy

**Affiliations:** Department of Animal Breeding and Genetics, Justus Liebig University, D-35390 Giessen, Germany; t.masthoff@rind-schwein.de

**Keywords:** rabbit, floor, dirtiness, pododermatitis, lesions

## Abstract

**Simple Summary:**

The German animal welfare requirements for rabbit housing did not take into account scientific results on the design of the floor. So, a maximum slot width of 11 mm and a degree of perforation of the raised level of 15% was required for growing rabbits. Such a floor was compared with alternative floor types in terms of the frequencies of polluted and injured animals. It could be shown that a floor following the requirements of the German animal protection ordinance led to the highest frequencies of polluted and injured animals compared with the other three floor designs. At the same time, alternatives for an animal-friendly floor, including the elevated platform, were presented under animal welfare aspects. The highest level of cleanliness and the lowest frequency of injured animals was demonstrated on a plastic floor with 5 mm slat width and 13 mm slot width, both on the base and on the elevated level (75% perforation).

**Abstract:**

In Germany, an animal welfare ordinance for the housing of rabbits was issued which did not take into account the current investigations on floor design. The aim of the investigations was to study the effects of floor design on dirtiness and occurrence of lesions on the legs of growing rabbits. A total of 1837 weaned rabbits, kept on four different floor designs, were examined for body lesions and the dirtiness of the soles of the feet at the end of the growing period. Two four-stage scoring systems (0–3) were used to record the dirtiness and the lesions on the feet. A floor according to the provisions of the German animal protection ordinance (10 mm slat width; 50% perforation on floor area; <15% perforation on the elevated platform) led to the most polluted and injured rabbits. The best cleanliness and the lowest injury rate of the growing rabbits was achieved on a plastic floor with 5 mm slat width and 13 mm slot width, both on the ground and elevated platform (75% perforation). The requirements of the German housing regulations on the floor for growing rabbits do not correspond to animal welfare.

## 1. Introduction

In intensive meat rabbit production, rabbits have been kept, until now, almost exclusively on wire mesh flooring which guarantees good hygienic conditions because of the separation from their droppings. In most cases metal mesh floors are used, with a diameter of the wire between 2 and 3 mm [1,2,3]. On wire floors, ulcerative pododermatitis is frequently observed in does [4]. Because of the does’ weight and the long time living under such conditions on wire-mesh floor, sore hocks could be very frequent [5,6]. Ulcerative pododermatitis causes chronic pain and suffering [4] as well as doe culling [6].

In growing rabbits, pododermatitis does not occur due to the short growing period, even when kept on wire mesh [1,7]. Trocino et al. [8] compared performance and behavior of growing rabbits kept on wire mesh or on cast iron slatted floor (2 cm slat and 1.6 cm slot width) and did not find differences. Trocino et al. [8,9] studied the possible influence of plastic slatted floor (1 cm slat, 0.7 cm slot width) versus the wooden slatted floor (8 cm slat, 3 cm slot width), and also of wire mesh (diameter of the wire: 2.5 mm, distance between wires: 1.5 cm) versus wire mesh with a layer of straw. Only the wooden floor and layer of straw resulted in a lower daily gain. Also, Abdelfattah et al. [10] did not find differences in growing performance and behavior between rabbits kept on wire mesh, plastic slatted floor (1.5 cm slot width), and slatted floor from rubber (1.5 cm slot width). Lang [11] and Wagner et al. [12] had already demonstrated that a plastic grid with a slot width of 13 mm and a slat width of 5 mm (perforation rate of approx. 75%) did not lead to serious lesions on the hind legs of growing rabbits. In 2014, the German animal protection ordinance was supplemented with requirements for rabbit keeping and, contrary to the scientific results [11,12], it was required that the slot width for growing rabbits should not exceed 11 mm and that the elevated platform should not be perforated to more than 15%. The aim of the investigations was to study the effects of floor design, especially on elevated platforms, following the German requirements on dirtiness and occurrence of lesions on legs of growing rabbits.

## 2. Materials and Methods

All procedures were performed in accordance with the guiding principles for the care and use of animals in research facilities [13].

The study was conducted on the research station “Oberer Hardthof” of the University of Giessen. In 12 rounds of growing, a total of 1837 weaned rabbits (ZIKA hybrids) were included. They were bought from a commercial breeder with an age of 35 days (age at weaning) and sold to the same rabbit breeder for slaughter after 8 weeks of housing. The housing took place in five pens and two large scale housing units (rabbit parks) in an air-conditioned chamber. This chamber has a ventilation system with supply and exhaust air tubes. The room can be heated and cooled (down to 18 °C) (Toshiba). The room was heated to 24 °C 24 h before the arrival of the weaned rabbits and after 14 days was set at a temperature of 20 °C (up to end of growing). The room was lighted from 6 am to 10 pm (150 lx). Natural lighting was possible via two windows in the anteroom, and the chamber had windows in the wall to the anteroom. Water was applied ad libitum by nipple drinkers (five per pen and eight per rabbit park). The drinking water was acidified with a mixture of organic acids (formic acid, propionic acid, lactic acid) to the pH of 4.5 to 5 in order to perform a hygienization of the water and to stabilize the gastrointestinal health of the rabbits.

The rabbit parks had a floor area with 30,000 cm^2^ (285 × 105 cm) each, with an elevated platform at a height of 38 cm measuring 10,830 cm^2^ (285 × 38 cm). The elevated platform was 32 cm from the rear wall, so that the rabbits could jump from the front and from the back to the second floor. The front wall of the park could be swung up the full width for easy work. The side walls were 80 cm high. The park was open at the top. The ground floor of the park was installed at a height of 100 cm. Feeding was possible from six feeders per park fixed on the ground floor with two feeding places each. The feeders were filled manually every day. Hay from grass was given ad libitum by two hay racks per park installed on the elevated platform.

The five identical enriched flat deck units had a floor area with 10,000 cm^2^ (100 × 100 cm) each, with an elevated platform at a height of 25 cm measuring 3000 cm^2^ (100 × 30 cm). The flat decks served as a control group. The side walls were made from galvanized sheet steel. The flat decks had a roof of wire mesh (2 × 4 cm mesh). The height of housing system was 50 cm. The working level was 100 cm for easy work. The feeder was installed at the front side and had four feeding places. The feeder could be filled from the front without opening the flat deck. The elevated platform was located directly on the back wall so that the animals could only jump on it from the front. One hay rack on the elevated platform was available in each flat deck (grass hay ad lib.).

**Figure 1:**
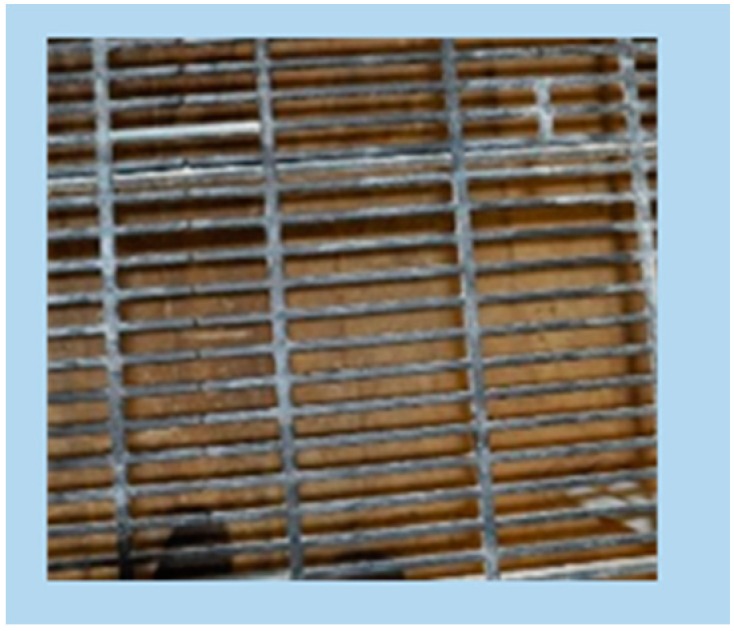
Plastic slatted floor with 5 mm slat width, 13 mm slot width, and 75% perforation—A (commercial plastic slatted floor for poultry, Siepmann, Herdecke, Germany).

**Figure 2:**
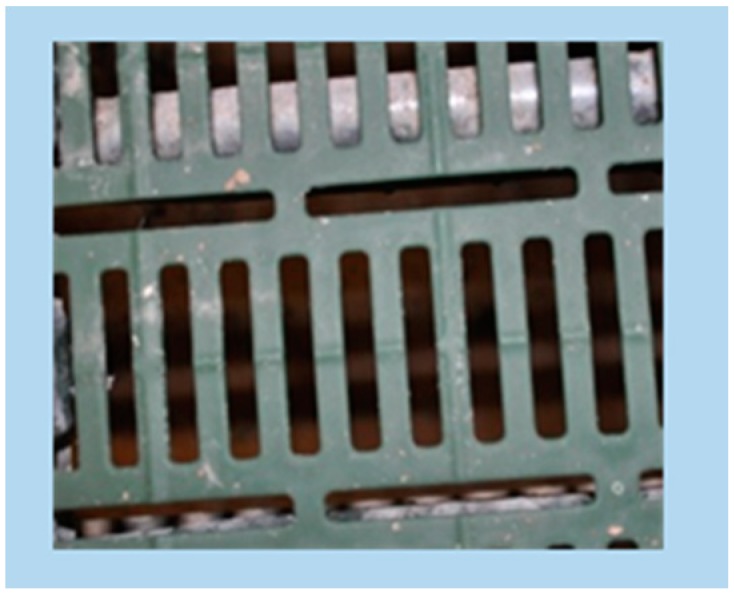
Plastic slatted floor with 10 mm slat width, 10 mm slot width, and 50% perforation—B (commercial plastic slatted floor for pigs, MIK, Siershahn, Germany).

**Figure 3:**
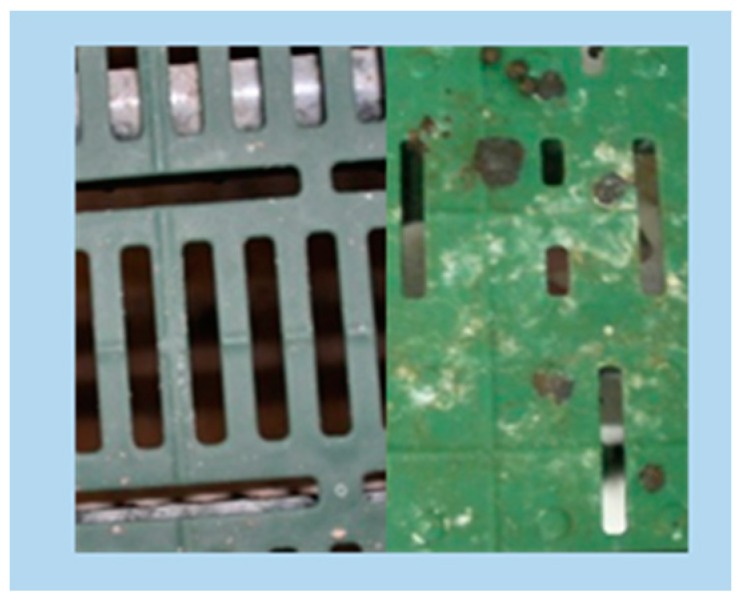
Plastic slatted floor with 10 mm slat width, 10 mm slot width, and 50% perforation on base floor area (like B) and floor with <15% perforation on the elevated platform—C (so called eco plastic slatted floor for pigs, MIK, Siershahn, Germany).

**Figure 4:**
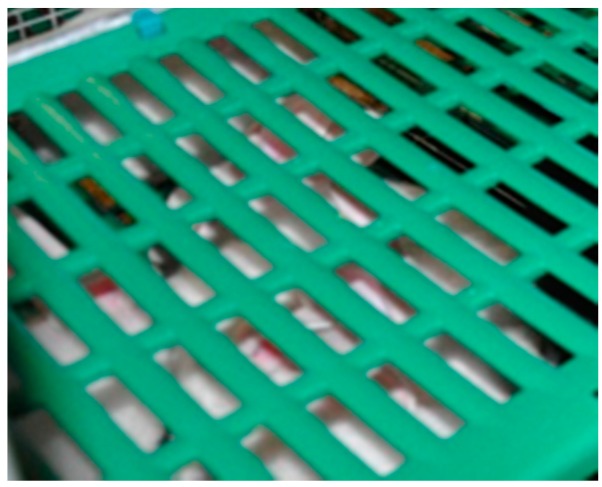
Plastic slatted floor with 12 mm slat width, 12 mm slot width, and 50% perforation—D (Meneghin, Povegliano, Italy).

The growing period was divided into two sections (pre growing with two and final growing with six weeks). All of the rabbits received a standard rabbit growing diet with a coccidiostat (diclazuril 1 ppm) in the first two weeks of the growing period with 15.0% crude protein and 16.3% crude fibre. Between the first and second period, the feed was changed over 5 days to a final growing diet (15.0% crude protein, 17.3% crude fibre). Four different floor designs were tested in this study:

**A** plastic slatted floor with 5 mm slat width, 13 mm slot width, and 75% perforation on both the floor area and the elevated platform (commercial plastic slatted floor for poultry, Siepmann, Herdecke, Germany) (Figure 1);

**B** plastic slatted floor with 10 mm slat width, 10 mm slot width, and 50% perforation on both the floor area and the elevated platform (commercial plastic slatted floor for pigs, MIK, Siershahn city, Germany) (Figure 2);

**C** slatted floor with 10 mm slat width, 10 mm slot width, and 50% perforation on the base floor area (commercial plastic slatted floor for pigs, MIK Siershahn, Germany) and floor with <15% perforation on the elevated platform (so called eco plastic slatted floor for pigs, MIK Siershahn, Germany) (Figure 3);

**D** slatted floor with 12 mm slat width, 12 mm slot width, and 50% perforation on both the floor area and the elevated platform (Meneghin, Povegliano, Italy) (Figure 4).

A floor design exactly meeting the requirements of German animal protection ordinance is not available on market. This is the reason why a floor with 10% and not with 15% perforation on elevated platforms was used. The floor of variant D is not permitted according to current German regulations (as the slot width is too wide).

**Figure 5:**
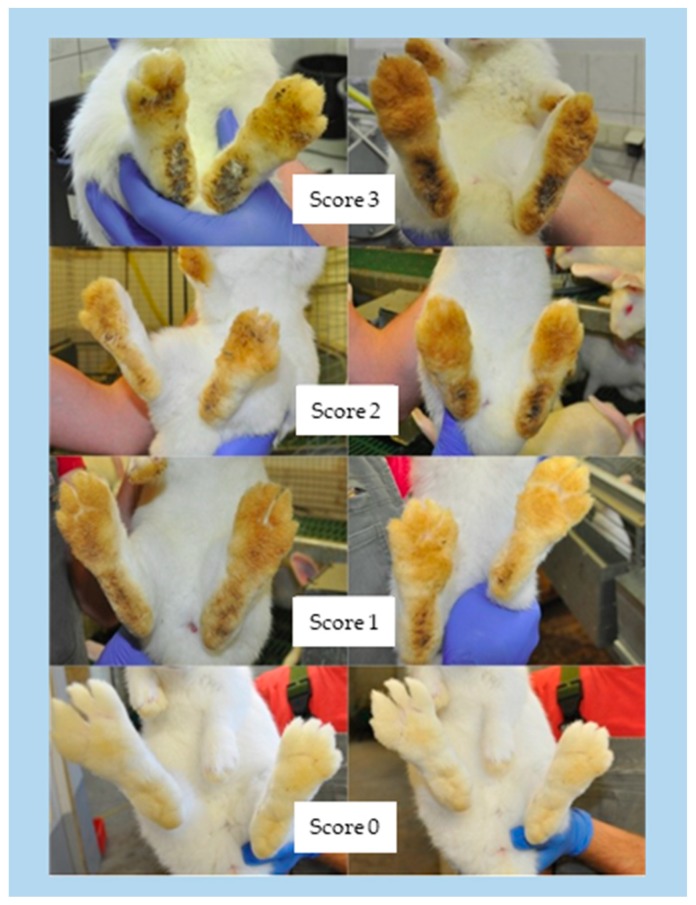
Pollution rating scheme with score 0 = clean to score 3 = heavily polluted.

**Figure 6:**
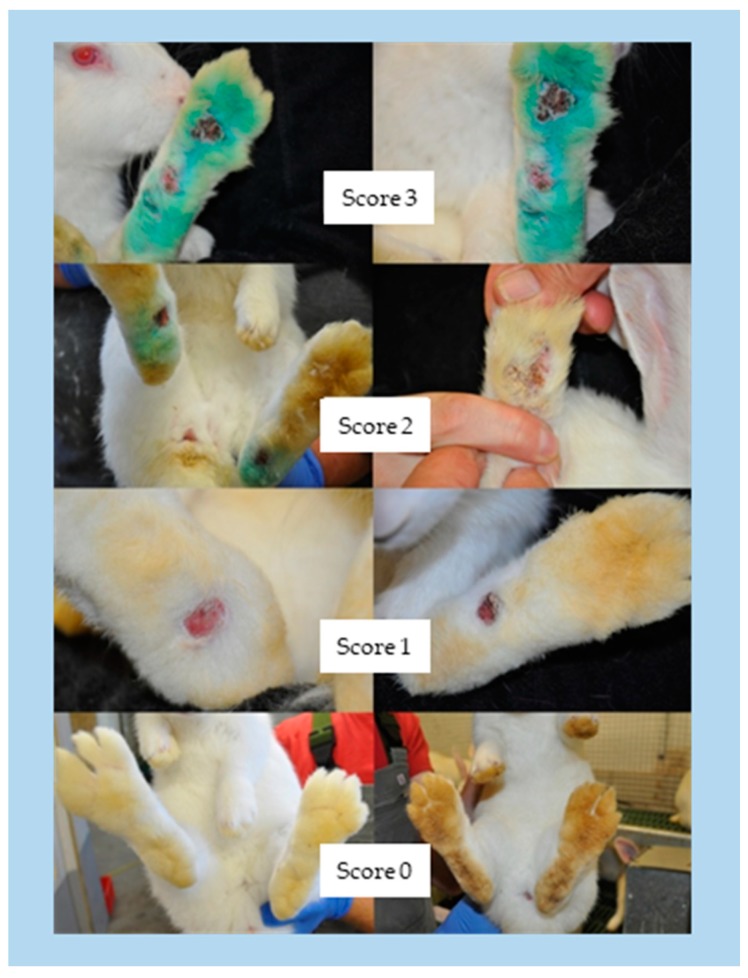
Scheme for assessing injuries to the (hind) limbs with score 0 = not injured to score 3 = severely injured.

At the beginning of each round, the weaned rabbits were weighed, sexed, and randomly placed in the seven housing units. At the end of the growing period the rabbits were weighed, examined for body lesions, and the dirtiness of the soles of the feet was scored. Two four-stage scoring systems (0–3) were used to record the dirtiness and the lesions on the feet. An unsoiled and uninjured condition was represented by zero. Concerning dirtiness, score 1 (+) describes a light soiling with small areas of faeces bonded especially in the bale area. Score 2 (++) characterizes medium pollution with a compact fouling of faeces and hair, and score 3 (+++) applies to large-scale contamination over the entire sole of the foot (Figure 5). Concerning lesions, score 1 (+) describes single circular hyperkeratosis or open wounds. Score 2 (++) is characterized by multiple circular injuries or single major injuries. Score 3 (+++) refers to large, open wounds that are deep and partially associated with abscesses (Figure 6, Masthoff [14]).

Statistical processing was carried out with the statistical program package IBM SPSS Version 23. First, and at the same time as a plausibility test, the descriptive statistics were calculated. To compare the percentages of contaminated or injured animals at the end of the growing period, the Chi square independence test was used in contingency tables. The significance was defined as *p* < 0.05.

## 3. Results

### 3.1. Frequency of Polluted Rabbits in Dependence on Floor Design

**Figure 7:**
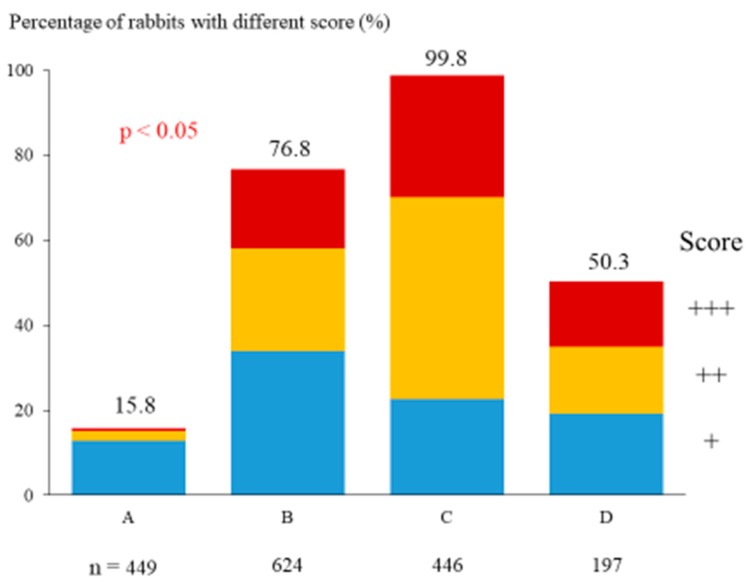
Influence of floor design on frequency of polluted rabbits at the end of the growing period.

For a floor according to the requirements of German animal protection ordinance (perforation of the elevated level = 10%, floor design C), the highest rate of polluted animals was recorded (99.8% of all animals scored). In total, 47.5% of the rabbits had a medium level and 28.7% had a high level of pollution. On the other hand, housing growing rabbits on a floor with a perforation rate of 75% both on the base and at the elevated level (floor design A) led to the lowest frequency of polluted rabbits. In total, 15.8% of the animals (out of 449) kept on A were polluted mostly on the hind legs (12.9%) at the end of growing (Figure 7). Housing growing rabbits on a floor with slatted floor with both 10 mm slat and slot width and 50% perforation on both the floor area and elevated platform (floor design B) led to the second highest percentage of polluted rabbits (76.8%), including 24.0% animals with a medium level of pollution and 18.8% with a high level of pollution. Rabbits kept on a newly developed plastic slatted floor with both 12 mm slat and slot width and 50% perforation on both the floor area and elevated platform had the second lowest percentage of polluted animals at the end of growing period (50.3%). 15.2% of rabbits were very dirty and 15.7% were polluted on a medium level (Figure 7). 

### 3.2. Frequency of Wounded Rabbits in Dependence on Floor Design

**Figure 8:**
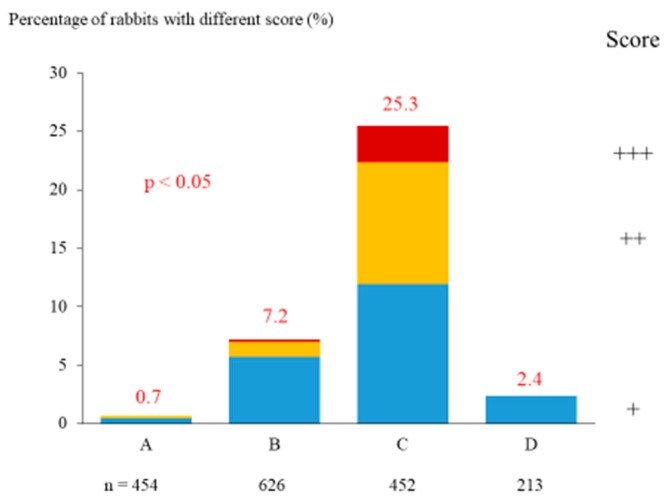
Influence of floor design on frequency of wounded rabbits at the end of the growing period.

From the point of view of animal health and animal welfare, a floor with 50% perforation on the floor and 10% perforation on the elevated platform (floor design C according to German animal protection ordinance) was by far the most problematic. This floor resulted in the statistically highest percentage of injuries (25.3%), mostly on the hind legs. 10.4% of rabbits had moderate lesions and 3.1% had severe lesions. The lowest rate of injured animals was found on a floor with 75% perforation both on the base level and elevated platform (floor design A with 0.7% of mild to moderately injured animals, *p* < 0.05) (Figure 8). Rabbits kept on this floor had no severe lesions and only 0.2% had moderate lesions on the hind legs. The housing of growing rabbits on a slatted floor with both 10 mm slat and slot width and 50% perforation on both the floor area and elevated platform (commercial plastic slatted floor for pigs—floor design B) led to a frequency of 7.2% wounded animals including 0.16% of rabbits with severe lesions and 1.3% with moderate wounds. The floor design D with slatted floor with both 12 mm slat and slot width and 50% perforation on both the floor area and the elevated platform resulted in the second lowest frequency of wounded rabbits. Only 2.4% of rabbits had mild lesions on their hind legs (Figure 8).

## 4. Discussion

The flooring design is a key factor for rabbit comfort and housing hygiene. Perforated floors in rabbit housing have the task of quickly separating animals from faeces and urine [2]. Therefore, the width of the slots and slats, the degree of perforation of the floor, and the floor material have great importance. If the degree of perforation is too low and the slots are not wide enough, the faecal pellets cannot fall through unhindered. As a result, the floor and the animals kept on it are polluted [12]. At the same time, the slots should not be too wide, so that the animals can move freely and are not getting injured [8]. A potential problem of using alternative floors with plastic grids or footpads is that the relatively large closed surfaces limit the self-cleaning of the floors and that can lead to greater pollution of the floors. The contamination of the floor cannot be measured directly, therefore, in the present study, the pollution of the hind legs of the rabbits was scored, which is the result of a more or less polluted floor.

The results of the evaluation of the degree of soiling of the hind legs showed that the rabbits kept on floor design A with plastic slatted floor with 5 mm slat width, 13 mm slot width, and 75% perforation on both the floor area and elevated platform (commercial plastic slatted floor for poultry) were the cleanest. In contrast, a floor according to the provisions of the German animal protection ordinance (floor design C: Plastic slatted floor with both 10 mm slat and slot width and 50% perforation on the base level and with <15% perforation on the elevated platform—eco plastic slatted floor for pigs) led to the most polluted animals. It should be underlined that the perforation level of the elevated platform (10%) was less than required (15%), as such a floor that meets the requirements of the law is not available on the market. Such a dirty floor should be considered as non-animal-friendly [15]. This evaluation is supported by the results of Morisse et al. [16] and Dal Bosco et al. [17]. Both groups of authors reported that, in choice tests, rabbits avoided heavily soiled areas and preferred clean metal grid floors compared to a littered area. When rabbits are kept in a polluted housing environment, the animals respond with increased brushing behavior to keep their fur clean [9,17].

From the point of view of animal welfare, the frequency of injured animals is more important than the percentage of polluted rabbits. In the present study, cases of pododermatitis were detected on floor designs B and C. This is surprising in that it is reported in the literature that pododermatitis does not occur in the housing of growing rabbits due to the short duration of the growing period [1,7]. Two possible causes for the occurrence of pododermatitis are discussed in literature. On the one hand, a strong punctiform loading of the hind legs, as can occur in the case of housing does on metal mesh, can lead to lesions [1,2,5]. On the other hand, the sticking of feet with faeces and the moistening with urine in the case of poor drainage of the floor is a problem [18]. In our own investigations, the reason for the high frequency of wounded rabbits kept on floor design C with the low percentage of perforation on the elevated platform is obviously the pollution of the floor [14]. This floor was heavily polluted, especially on the platform, leading to a very high percentage of polluted rabbits (Figure 7). The floor design C, which meets the requirements of the German animal protection ordinance, is not suitable for rabbits, because a high proportion of injured animals occurs, which would be avoidable with appropriate design of the floor, especially of the elevated level. Above all, a degree of perforation of 15 or 10% on the elevated platform leads to heavy pollution and consequently to injuries of the growing rabbits. The prevalence rate of leg lesions in the growing rabbits on this floor is almost twice that reported by Rosell and De La Fuente [4] for does (13.7%). Ulcerative pododermatitis causes chronic pain and suffering [4] and such a floor does not meet the requirements for animal-friendly housing [15].

The floor design A, made of plastic, with a degree of perforation of 75%, a slot width of 13 mm, and a slat width of 5 mm is suitable for animal use in view of the requirements defined by Hoy et al. [19] (separation of animals from excrements, unavoidable low morbidity rate). However, this optimally functioning floor is no longer allowed due to (allegedly) too wide slots and too high perforation on an elevated platform if the requirements of the German animal protection ordinance have to be complied with. Floor design B, with plastic slatted floor, with 50% perforation on both the floor area and the elevated platform (commercial plastic slatted floor for pigs) has 10 mm wide, straight bars, on which the moisture cannot drain so well, so that the relatively high rate of pollution and injury can be explained. Floor design D (slatted floor with both 12 mm slat and slot width and 50% perforation on both the floor area and elevated platform) resulted in the second lowest percentage of polluted and injured rabbits at the end of the growing period. The surface of the floor is slightly curved so that urine can flow off better. The floor dries quickly, the animals are relatively clean and little, and only slightly injured. According to the provisions of the German animal protection ordinance, this floor is not allowed because the slots are about 1 mm wider than required.

## 5. Conclusions

The floor design and degree of perforation of the elevated platform, as prescribed in the German animal protection ordinance, are not suitable for rabbits, as they lead to a comparatively high rate of injured animals. The best cleanliness and the lowest injury rate of the growing rabbits were achieved on a plastic slatted floor with 5 mm slat width and 13 mm slot width (75% perforation). However, this floor design is not allowed anymore in Germany since 2019. A floor that complies with German animal welfare legislation is not yet on the market.
